# Implanting totally implantable venous access port via the internal jugular vein guided by ultrasonography is feasible and safe in patients with breast cancer

**DOI:** 10.1186/1477-7819-12-378

**Published:** 2014-12-08

**Authors:** Jie Zhou, Shikun Qian, Weixing He, Guodong Han, Hongsheng Li, Rongcheng Luo

**Affiliations:** Cancer Center, Nanfang Hospital, Southern Medical University, No. 1838, Northern Guangzhou Road, 510515 Guangzhou, Guangdong China; Department of Breast Oncology, Affiliated Cancer Hospital of Guangzhou Medical University, No. 78 HengZhiGang Road, Yuexiu, 510095 Guangzhou, Guangdong China; Department of Hepatobiliary Surgery, The Second Affiliated Hospital of Guangzhou Medical University, No. 250 ChangGang Middle Road, Haizhu, 510260 Guangzhou, Guangdong China

**Keywords:** Internal jugular vein, Totally implantable venous access port, Ultrasonography

## Abstract

**Background:**

Because of long-term use for chemotherapy and fluid administration in cancer patients, a totally implantable venous access port (TIVAP) has been advised as a feasible catheter. The purpose of this study was to evaluate the effectiveness and safety of ultrasound (US)-guided internal jugular vein (IJV) puncture for TIVAP implantation in patients with breast cancer.

**Methods:**

We reviewed the medical records of 492 patients who underwent US-guided IJV puncture for TIVAP implantation at our oncology department between 2010 and 2013. Indications, surgical complications, and early and long-term complications were analyzed.

**Results:**

All TIVAPs were implanted successfully. Indications for TIVAP were chemotherapy alone (88 patients), chemoradiotherapy (387 patients), surgery (12 patients), and parenteral nutrition (5 patients). Complications were observed in 65 (13.21%) patients. The median duration of the TIVAP was 359 days (range, 28 to 712 days) without damage to the port or catheter, or leakage of drugs outside of the port system.

**Conclusions:**

A TIVAP can be employed for chemotherapy and parenteral nutrition on the implantation day. Using a US-guided IJV puncture to completely implant a TIVAP is feasible and safe in patients with breast cancer.

## Background

A totally implantable venous access port (TIVAP) provides a simple and safe means of accessing the vascular system for intravenous delivery of chemotherapeutic drugs and supportive care, which are widely used in clinical oncology [1, 2]. In the treatment of breast cancer, chemotherapy regimens with a duration of up to 6 months are commonly required [3]. Secure venous access is desirable for these treatments as chemotherapy drugs are toxic to the veins [4]. In our department, anthracycline- and docetaxel-containing regimens are frequently used as first-line treatments for breast cancer patients. Therefore, the placement of a TIVAP at the beginning of a chemotherapy regimen with potentially necrosis-inducing agents is required [5]. A TIVAP can be implanted via the basilic vein, subclavian vein, external jugular vein, or the internal jugular vein (IJV). Since patients with breast cancer must undergo ipsilateral radiotherapy and blood pressure measurements or since they may carry heavy objects using the contralateral arm, the IJV and subclavian vein of the contralateral side are the suitable choices for TIVAP placement. However, puncturing of the subclavian vein is associated with considerable complications, including pneumothorax, hemothorax, injury of large vessels, and catheter pinch-off within the costoclavicular space [1, 6]. Thus, the IJV is considered as the primary effective vein.

From January 2010, the TIVAP via IJV approach has been used in our institution and the TIVAP is used immediately on the implantation day. A port TIVAP is implanted into the healthy side, and the catheter is introduced via the IJV to the superior vena cava (SVC). Herein, we report on our observations associated with the use of a TIVAP implanted through ultrasound (US)-guided IJV puncture.

## Methods

### Patients

This was a retrospective study including 492 patients with breast cancer. Patient demographics and characteristics are shown in Table 1. Of the 492 patients, 5 were male and 487 were female; they had a median age of 48.66 years (range, 22–73 years). All patients underwent fluid administration and/or chemotherapy. Between January 2010 and December 2013, all patients underwent TIVAP placement via the IJV or subclavian vein to the SVC. All patients were followed-up until TIVAP removal, death, or hospital discharge. The procedure and its possible complications were explained to patients, and written informed consent was obtained.

**Table 1:** **Demographics of the study population (n = 492)**

Characteristics	Value	Percentage
Median age (years)	48.66 ± 11.05	
Age range (years)	22–73	
**BMI, kg/m** ^**2**^		
>24	367	74.59
≤24	125	25.41
Median BMI	24.72 ± 2.72	
**Male/Female ratio**	5/487	1.02/98.98
**Breast cancer**		
Right/Left	236/256	47.97/52.03
**Puncture site**		
Right internal jugular vein	251	51.02
Left internal jugular vein	229	46.54
Right subclavian vein	5	1.02
Left subclavian vein	7	1.42
**Treatment**		
Chemoradiotherapy	387	78.66
Chemotherapy alone	88	17.89
Surgery	12	2.44
Nutritional therapy	5	1.02
**Follow-up period, days**		
Median days of the catheter	359.13 ± 183.88	
Range	28–712	
Total catheter days	176,694	

### Main equipment

The port system had two components: a silicone intermediate-sized reservoir with a large central injection septum made of silicone rubber and a polyurethane catheter with a Groshong® valve (Bard Port isp Access System, Salt Lake City, USA).

### Implantation procedures

Prior to surgical implantation, blood count, prothrombin time, and routine biochemistry parameters were obtained. Patients were placed in a supine position on the operative table. Catheter length was measured according to body surface prior to the procedure. Local anesthesia was administrated subcutaneously, restricted to the area of the port implantation and the venous puncture. No routine prophylactic antibiotics were administered. A standard surgical sterile technique was employed in all cases, including a surgical scrub.

The IJV was the preferred access site. The subclavian vein was the second choice for the surgical procedure. After local anesthetization with 1% lidocaine, the IJV was punctured under direct ultrasound visualization (10 MHz ultrasound transducer, Sonosite S series, Bothell, WA, USA) using an 18-gauge needle. A subcutaneous pocket was created on the contralateral side of the anterior chest wall while avoiding any possible radiation fields. A 2-cm skin incision was made with an 11-blade scalpel and anesthetized with 1% lidocaine. The subcutaneous tissues of the port pocket and tunnel were anesthetized with 1% procaine with epinephrine (1:200,000). Through a blunt and sharp dissection, the port pocket was created. Using a tunneling device, the catheter was tunneled from the pocket incision to the IJV puncture site. A port was connected to the indwelling catheter through a subcutaneous tunnel and was implanted in the pocket. The wound was closed with a 4-0 resorbable suture. X-ray surveillance was subsequently used for the silicone rubber and polyurethane catheter placement. The catheter tip was positioned at the junction of the right atrium to the SVC.

**Figure 1:**
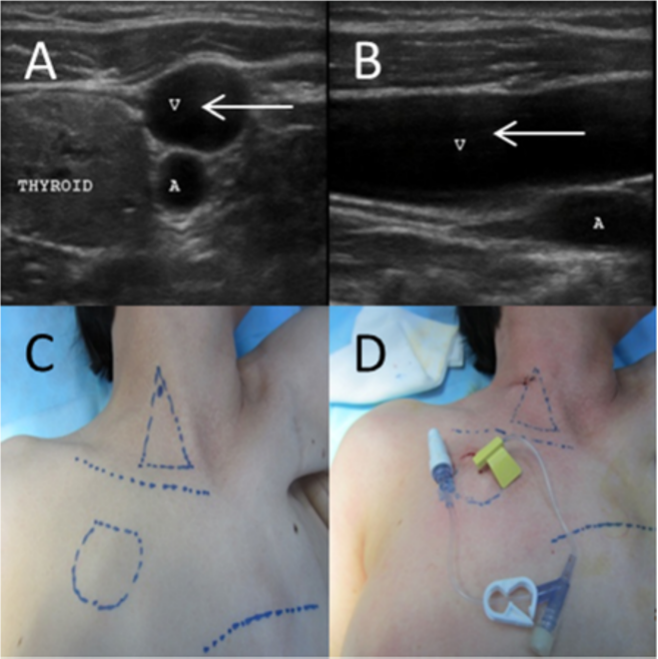
**TIVAP implantation procedure.** US-guided IJV (V) surgical procedure **(A,** **B)**. Lines were drawn to create the anatomical landmark, surgical incision, and puncture site **(C)**. Insertion of a continuous infusion needle **(D)**.

**Figure 2:**
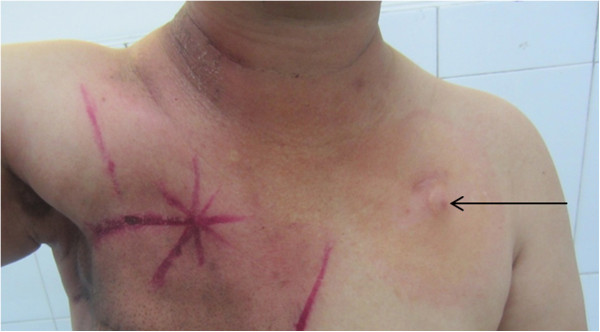
**The TIVAP was implanted into the contralateral side to avoid the radiotherapy area.**

**Figure 3:**
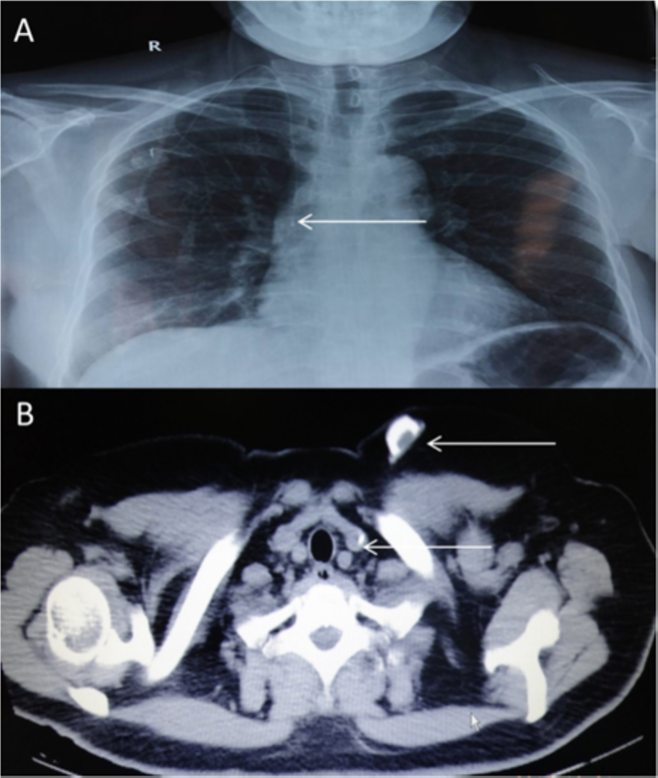
**Catheter tip and port location.** X-ray examination for catheter tip location **(A)**. CT showing location of port and catheter **(B)**.

For patients who required continuous infusion, the needle and infusion line set were changed every week. A total of 10 mL of heparinized saline solution (100 IU/mL) was administered as a flush solution before needle removal (Figures 1, 2 and 3).

### Patient follow-up

TIVAP implantation was performed in breast cancer patients who required a large number of chemotherapy regimens and cycles and/or fluid administration. The early and late complications were all observed and recorded from the day of implantation procedure to December 2013. Variables were recorded as medians and the range was given. In the 492 patients, the median duration of TIVAP use was 359 days (range, 28–712 days) without any break to the port system or any leakage of drugs.

### Statistical analysis

Statistical analysis was conducted using the SPSS 19.0 statistical analysis package. The data were analyzed using Kaplan-Meier survival analysis.

## Results

### Patient characteristics

TIVAP implantation was performed for chemotherapy alone (88 patients), chemoradiotherapy (387 patients), surgery (12 patients), or parenteral nutrition (5 patients). In total, 176,694 catheter days (average 359 days per patient; range, 28–712 days) were analyzed. Of these 492 patients, 17 patients did not receive their planned intravenous chemotherapy and the TIVAPs were only effectively used for surgery or parenteral nutrition. In the remaining patients, the median number of chemotherapeutic cycles after TIVAP implantation was 5 (range, 2–8). Right breast cancer was observed in 236 patients and left breast cancer in 256 patients. The median time for the implantation was 32 min (range, 23–50 min).

### Complications

**Figure 4:**
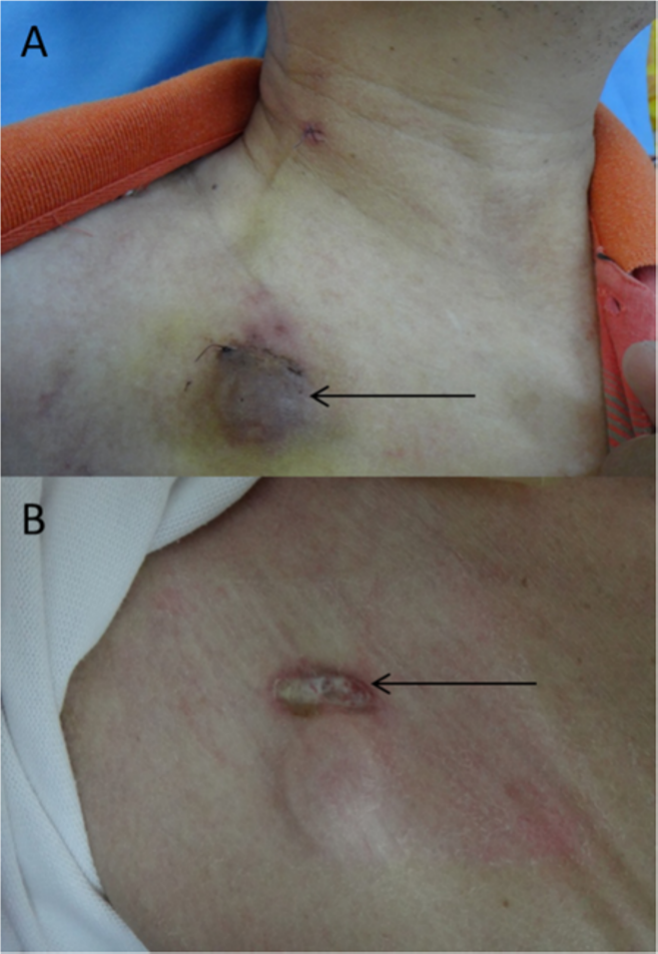
**Complications of hematoma (A) and infection (B).**

The early complications are summarized in Table 2. Hematoma was observed in 12 patients (in 7 patients this occurred in the implantation site and in 5 patients in the implanted pocket) and resolved within 2 weeks (Figure 4A). Early infection was observed in 2 patients (Figure 4B). Cardiac arrhythmia occurred in 10 patients, probably due to the catheter tip touching the right atrium. After replacing the catheter tip on the SVC or changing body position, the arrhythmia disappeared.

**Table 2:** **Early complications**

Early complications	n = 492	Percentage
Hematoma	12	2.44
Cardiac arrhythmia	10	2.03
Arterial puncture	6	1.22
Guide wire bending	3	0.61
Bleeding	2	0.41
Introductory sheath kinking	2	0.41
Pock early infection	2	0.41
Total	37	7.52

The late complications are summarized in Table 3. Catheter-associated thrombosis was observed in 12 patients (2.44%) approximately 12 weeks after implantation. These patients received warfarin and heparin treatment and their TIVAP continued to be used without further complications. Pulmonary thromboembolism was not observed in any patients. Catheter migration and embolization was documented in 6 patients. Port pocket infection due to cutting of the skin with the needle during repeated puncture was observed in 3 patients (0.61%) after 3 months of use for chemotherapy and fluid administration. Among the 12 patients who were converted to the subclavian vein, 3 were diagnosed with pinch-off syndrome.

**Table 3:** **Late complications**

Late complications	n = 492	Percentage
Catheter-associated venous thrombosis	12	2.44
Catheter migration and embolization	6	1.22
Catheter-related infection	4	0.81
Port pocket infection	3	0.61
Pinch-off syndrome	3	0.61
Total	28	5.69

### Patient follow-up

Reasons for TIVAP removal are shown in Table 4. The TIVAPs of 322 patients were removed at the end of therapy. Catheter occlusion led to the removal of TIVAP from 17 patients, 4 removals were due to incurable infection with bacteremia due to penicillin-resistant staphylococcus aureus, and 1 patient (0.20%) died within 1 month of implantation because of disease progression.

**Table 4:** **Reasons for removal of TIVAP (n = 492)**

Reason	n	Percentage
End of therapy	322	65.45
Catheter occlusion	17	3.46
Infection	4	0.81
Death	1	0.20

## Discussion

The totally implantable port system was introduced to the clinic by Dr. Niederhuber in 1982. The main purpose of a TIVAP in clinical practice is to provide a secure and comfortable route for cytotoxic drugs for patients with malignancies and intravenous parenteral nutrition solutions [1, 7]. In this study, we evaluated the consequences of using a TIVAP in patients with breast malignancy, since there were only a few reports on the use of a TIVAP in the Chinese population or regarding the US-guided implantation approach through the IJV [5].

The IJV is located at the apex of the triangle formed by the heads of the sternocleidomastoid muscle and the clavicle. The subclavian vein crosses under the clavicle just medial to the midclavicular point [8]. These veins are invisible on the skin surface, and were therefore originally blindly punctured based on the operator’s experience. Image guidance can improve the accuracy of vascular puncture and virtually eliminates the risk of several complications reported with unguided placement such as pneumothorax, hemothorax, and nerve injury [9]. None of these complications occurred in patients who underwent US-guided placement. Under US visualization, the introducer needle is guided through the skin and into the vein. The IJV is difficult in obese patients and therefore conversion to the subclavian venous vein for TIVAP implantation is indispensable. In our department, all TIVAPs were implanted via the IJV or subclavian vein successfully. After implantation, chemotherapy and/or fluid administration were performed on the same day.

Cardiac arrhythmia and hematoma are the most common mechanical complications during the insertion of central venous catheters. These two complications were observed in 22 (4.47%) patients, which is lower than in previous studies [5, 10]. Cardiac arrhythmia is avoidable with careful catheter placement. Hematoma may be caused by incorrect artery puncture or abnormal menstrual periods in breast cancer patients. Local sufficient compression for 10 to 15 minutes immediately stops bleeding. With respect to guide wire bending and introductory sheath kinking, we experienced two patients requiring intraoperative conversion from the IJV to the subclavian vein. Since one patient died early after the implantation of the TIVAP, for causes unrelated to TIVAP implantation, good performance status and sufficient organ function to receive antitumor treatment were verified before the implantation procedure in order to consider the benefit for patients from TIVAP. The early infection within the pocket was observed in 2 patients, probably due to bacteremia from the incision or needle site, which is consistent with prior reports [10, 11]. Application of antibiotic ointments and oral antibiotic drugs were advised.

Thromboembolic complications are the major late potential problem in the long-term management of TIVAPs. Catheter implantation itself carries a risk of venous thrombosis [1, 12]. In our clinical observation, catheter-associated venous thrombosis was recorded in 12 (2.44%) patients. The incidence in previous reports ranges from 0.3% to 23% [5, 7, 11, 13–18]. Vescia et al. reported that low molecular weight heparin may be ineffective and that a higher dose may result in a positive outcome [1]. Karthaus reported that in 439 cancer patients with a central venous catheter, dalteparin prophylaxis did not reduce the frequency of thromboembolic complications [19]. Sutherland found that the prompt use of anticoagulation therapy after the occurrence of venous thrombosis is essential [12]. Ahn observed that a diluted heparin solution flushed every month is essential [13]; the incidence of thrombosis in their study was lower than in ours, probably as a result from the different catheter material and structure [13]. When catheter-related thrombosis was observed, anticoagulant therapy was administrated first in the current study; if thrombolytic therapy was efficient, immediate removal of the port was not necessary. Catheter tip thrombosis with the port system is flushable but there is no aspiration of blood via the tip. In our department, 10,000 IU heparin over 24 hours is flushed into the port system for 3 days via a perfusion system, after which treatment the port system often becomes effective again.

Catheter migration and embolization were recorded in 6 (1.22%) patients, which is lower than that reported in previous studies [20], probably due to avoiding excessive movement and the use of a Groshong® valve catheter in our department.

Catheter-related infection is also a potential long-term complication in the management of a TIVAP [5], and its diagnosis if often difficult in the absence of local signs of inflammation [21]. In our study, the complication was observed in 4 (0.81%) patients, which was less than the rate reported in the literature [7, 10, 11, 22–25]. Paired blood cultures (aerobic and anaerobic) from a peripheral vein and the central catheter were conducted to evaluate the possibility of bacteremia. A positive culture of blood from a central venous catheter indicates contamination of catheter colonization or a catheter-related bloodstream infection. Patients with a negative result require a second culture for confirmation. Port systems must be removed in case of persistent bacteremia infection after antibiotic treatment.

In our study, 3 (0.61%) patients with pinch-off syndrome were observed. Their catheters were placed between the clavicle and the first rib via subclavian vein access. Nikolaos et al. reported a complication rate of 2.5% [10], which was higher than that in our study. The variation could be caused by the difference in implantation site.

**Figure 5:**
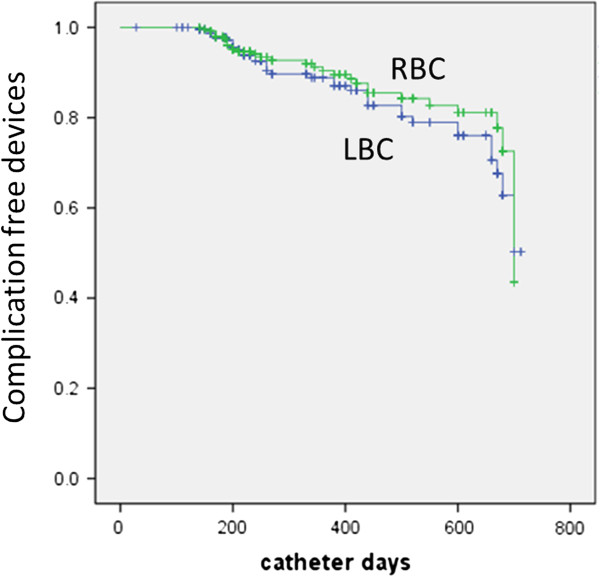
**Kaplan-Meier survival curve showing complication-free catheter duration over time for both study groups.** RBC, complication of right breast cancer; LBC, complication of left breast cancer.

The distance between the left IJV or the left subclavian vein to the junction of the right atrium and SVC is greater than on the right side. The catheter length inserted into the body from the left side is longer than that from the right, which may cause more complications. However, in our study, the difference between the complications of right and left sides was not significant (Figure 5).

The timing of TIVAP use is controversial. Nuriye et al. reported that chemotherapy administration immediately after a venous port catheter implantation appears safe without increased acute and chronic complications in an inpatient setting [17]. However, Narducci et al. found that a minimal interval of 8 days between placement of the TIVAP and its first use can reduce complications [20]. In our study, the overall complication rate of 13.21% was similar to the 10 to 20% complication rate reported for several large surgical series in which both chest and arm ports were placed [9, 26]. Our results support clinical administration of TIVAP on the implantation day.

In our institution, we discuss with our patients the option of maintaining the port system implanted for up to 2 years after adjuvant breast cancer therapy owing to the increased risk of relapse within this period. The port systems may be needed again for palliative chemotherapy in metastatic patients [1]. Based on our experiences, the median time of catheter follow-up was 12 months by heparin flushing of the port system at 1-month intervals, similar to previous studies [27, 28]. No complications regarding a breakdown of the port system or drug leakage were noted during the observation period of 1 to 24 months in our study. Therefore, it is clear that careful and strict antiseptic handling can avoid contamination and minimize catheter-related infections.

## Conclusions

Antitumor therapy including chemoradiotherapy and supportive treatments have become more important for increasing overall survival and disease free survival in patients with breast cancer. To ensure long-term central venous access, the TIVAP has been extensively used and is now widely accepted as an effective catheter [29]. Our results show that TIVAP implantation via US-guided IJV is safe and feasible in the long-term use by breast cancer patients requiring chemotherapy and parenteral nutrition. Chemotherapy and/or fluid administration on the TIVAP implantation day appear safe without an increased risk of acute and chronic complications.

## Abbreviations

IJV: Internal jugular vein
SVC: Superior vena cava
TIVAP: Totally implantable venous access port
US: Ultrasound.
